# Improved hepatitis C screening and treatment in people who inject drugs should be a priority in Europe

**DOI:** 10.1186/1471-2334-14-S6-S3

**Published:** 2014-09-19

**Authors:** Achim Kautz, Lilyana Chavdarova, Margaret Walker

**Affiliations:** 1 European Liver Patients’ Association, Sint-Truiden, Belgium

## 

Since its launch in 2005, the European Liver Patients’ Association (ELPA) has worked to promote the interests of people with all forms of liver disease. Despite improvements in medical treatment, and despite the recent publication of WHO guidelines for screening, the overall situation of hepatitis C virus (HCV) transmission in Europe is a growing concern for ELPA. Many patients who are infected with HCV can carry the infection for many years before developing symptoms [1], and people who currently have chronic HCV will represent a heavy disease burden in the coming years [2]. The World Health Organization (WHO) estimates that more than 19 million people in Europe are infected with Hepatitis C, and states that transmission is mainly concentrated in people who inject drugs (PWID), who globally have 67% HCV prevalence [3].

Since there is no vaccine for Hepatitis C, prevention efforts must concentrate on health education programmes targeting high-risk populations and on public awareness campaigns that seek to improve testing and case finding in the general community [1].

On 1 December 2010, World AIDS Day, the European Monitoring Centre for Drugs and Drug Addiction published guidelines on testing for HIV, viral hepatitis and other infectious diseases in PWID. The new guidelines recommended a strategy to increase the uptake of testing among PWID in Europe and beyond. This would enable earlier treatment of infected individuals and reduce the further spread of the disease [4].

Two years later, the Euro Hepatitis Care Index called attention to the need for screening programs in risk populations to be improved in all countries, noting that in order to increase testing in drug users it is important to make the services as reachable and convenient as possible [1]. More recently, WHO recommended screening to identify persons with HCV infection, and also recommended that HCV testing be offered to individuals who have a history of HCV risk behaviour or who belong to populations with high HCV prevalence [3].

There is evidence that where HCV screening of PWID is implemented, it can be cost-effective [5] and screening programmes can be successful [6]. However it is not clear which countries have adopted these guidelines and there are still relatively few published reports on best practices.

Identifying infected patients through screening programmes is only part of the solution, and ELPA continues to be concerned about the slow introduction of new drugs for HCV treatment. The Euro Hepatitis Care Index showed that general access to the direct-acting antiviral drugs telaprevir and boceprevir only existed in five European countries, and that one-third of countries were not providing these new drugs at all [1]. WHO states that avoiding discrimination or stigmatisation of PWID is essential, and reports that treatment for HCV infection is both efficacious and cost-effective in PWID. WHO recommends that all adults and children with chronic HCV infection, including PWID, be assessed for antiviral treatment. WHO also notes that treatment may be effective as prevention due to a reduction in transmission [3].

In order to bring about change and to ensure that guidelines and effective treatment programmes are implemented in Europe, ELPA will continue to call for the reporting of additional data on HCV screening and treatment measures. We look forward to further changes being made in European policies and healthcare strategies which lead to success in tackling this chronic disease.

## Competing interests

The authors declare that they have no competing interests.

## Declarations

This article has been published as part of *BMC Infectious Diseases* Volume 14 Supplement 6, 2014: Viral Hepatitis in Europe. The full contents of the supplement are available online at http://www.biomedcentral.com/bmcinfectdis/supplements/14/S6. The publication charges for this supplement were funded by AbbVie as an unrestricted grant to Rigshospitalet, the University of Copenhagen. AbbVie further funded the printing of the supplement with additional financial support from the Drug Prevention and Information Programme (DPIP) of the European Union.
